# Extended High‐Dose Tofacitinib Induction Improves Clinical Response in Ulcerative Colitis: A Multicenter Cohort Study

**DOI:** 10.1002/jgh3.70410

**Published:** 2026-05-12

**Authors:** R. Gilmore, T. Pham, R. G. Fernandes, S. Lo, E. K. Wright, O. Rosella, P. De Cruz, B. Andrew, A. Vasudevan, K. Gazelakis, G. T. Moore, L. Thin, K. Au, S. Ghaly, C. Chen, W. Mohsen, T. Goodwin, M. P. Sparrow, M. G. Ward, M. Chew, M. Garg, E. Shelton, I. C. Lawrance, A. Thorat, C. da Fonseca Pereira, J. Begun, Y. K. An

**Affiliations:** ^1^ Department of Gastroenterology Mater Hospital Brisbane Australia; ^2^ Mater Research Institute, University of Queensland South Brisbane Australia; ^3^ Department of Gastroenterology St. Vincent's Hospital Melbourne Australia; ^4^ Department of Gastroenterology Austin Health Melbourne Australia; ^5^ Department of Gastroenterology Eastern Health Melbourne Australia; ^6^ Department of Gastroenterology Monash Health Melbourne Melbourne Australia; ^7^ School of Clinical Sciences, Monash University Melbourne Victoria Australia; ^8^ Department of Gastroenterology Fiona Stanley Hospital Perth Australia; ^9^ Department of Gastroenterology St Vincent's Hospital Sydney Australia; ^10^ Department of Gastroenterology Gold Coast University Hospital Gold Coast Australia; ^11^ Department of Gastroenterology Alfred Health and Monash University Melbourne Australia; ^12^ Department of Gastroenterology Northern Health Melbourne Australia; ^13^ Department of Gastroenterology Yarra Gastroenterology Melbourne Australia; ^14^ Faculty of Health and Medical Sciences University of Western Australia Crawley Australia; ^15^ Pfizer Australia Sydney New South Wales Australia

**Keywords:** extended induction, Janus Kinase inhibitor, tofacitinib, ulcerative colitis

## Abstract

**Background and Aims:**

Tofacitinib, an oral Janus Kinase (JAK) inhibitor, has demonstrated efficacy for ulcerative colitis (UC) in clinical trials, but the optimal dosing regimen remains unclear. This study aims to assess the effectiveness and safety of tofacitinib for UC in a real‐world Australian cohort focusing on outcomes of standard 8‐week induction with 10 mg twice daily compared with extended 16‐week induction.

**Methods:**

This multicenter study included patients with moderate‐to‐severe UC initiating tofacitinib between October 2020 and March 2021 across 13 Australian Inflammatory Bowel Disease centers. Clinical, biochemical, endoscopic, and adverse event (AE) outcomes were collected at baseline, week 8, week 16, and week 52, and stratified by duration of induction therapy.

**Results:**

A total of 124 patients (41% female; median age 41 [IQR 29–49] years) were identified, with 40% receiving standard induction and 60% receiving extended induction. Overall clinical response was achieved by 70% at week 8, 60% at week 16 and 68% at week 52. A significantly higher proportion of patients who received extended induction achieved clinical response by week 16 (69% vs. 45%, *p* = 0.02) and week 52 (75% vs. 54%, *p* = 0.03) compared with standard induction. Over 52 weeks, AEs and serious AEs were seen in 17% and 7% of patients, respectively. AEs of special interest occurred in 6% of patients (herpes zoster in 6/8 patients).

**Conclusions:**

Tofacitinib was effective in an UC real‐world Australian population and safety was consistent with its known profile. Clinical response at 16 and 52 weeks was significantly higher with extended induction dosing of tofacitinib.

## 1 Background and Aims

Janus Kinase (JAK) inhibitors are oral small molecule therapies that inhibit cytokine signaling through the signal transduction and activation of transcription (STAT) pathways [1]. Tofacitinib inhibits JAK 1–3 and is approved for the treatment of moderate to severe ulcerative colitis (UC) in Australia. Tofacitinib, the first JAK inhibitor to be approved for UC, demonstrated efficacy in inducing and maintaining remission in the phase 3 OCTAVE trials, with clinical response rates of 16%–18% by week 8 (Δ 10.3%–13% vs. placebo) and remission rates of 34%–40% (Δ 23%–29%) by week 52, showing significant benefit over placebo [2]. The OCTAVE open trial addressed patients who failed to respond clinically at week 8 to induction therapy with 10 mg of tofacitinib twice daily and were treated with a further 8 weeks of extended induction therapy [3]. Over 50% of these patients subsequently responded clinically at the end of week 16 with no significant difference in safety. However, no clear predictors were identified to guide targeted treatment for the subgroup with initial non‐response, nor was it evident whether extending induction therapy by a further 8 weeks might benefit all patients, including those already responsive at week 8. Subsequent long‐term extension efficacy and safety data from the registration trials over 7 years showed sustained remission in an ulcerative colitis population [4], with hundreds more patient years of safety data in a rheumatological population [5]. Concerns were raised about the safety of tofacitinib after the ORAL surveillance study showed increased risk of major adverse cardiac events and malignancy in an elderly rheumatological population with cardiac risk factors [6]. To date, these safety signals have not been replicated in a UC population. Tofacitinib appears to have a rapid mechanism of action regardless of disease severity, as shown by the reported case series in the most severely unwell UC patients with acute severe ulcerative colitis (ASUC) [7, 8, 9].

Despite the robust efficacy of tofacitinib in pivotal trials in UC, these controlled settings may not fully capture its effectiveness in routine clinical practice. Two large real‐world studies showed clinical remission rates above 30% at 8–12 weeks, and high rates of clinical response up to 70% which were sustained to week 52 [10, 11], yet generalizability is limited by regional variation in patient populations, dosing regimens, and access pathways. A further smaller study compared 52 weeks of high dose tofacitinib at 10 mg twice daily to standard 8 week induction and 5 mg twice daily ongoing, with no significant difference observed between the groups [12]. In Australia, tofacitinib is available as a first‐line advanced drug therapy (ADT) without prior ADT failure, providing a unique opportunity to evaluate its effectiveness in ADT‐naïve and exposed patients.

This study aims to assess the real‐world effectiveness and safety of tofacitinib in UC patients receiving early access therapy in Australia, focusing on the duration of induction therapy and associated clinical outcomes in routine clinical practice.

## 2 Methods

### 2.1 Study Design

This national, multicenter, retrospective cohort study evaluated the use of tofacitinib for moderate to severe UC in Australia. The study included patients from 13 inflammatory bowel disease centers who commenced tofacitinib for UC between February 2019 and July 2022 via the patient familiarization program (PFP). Eligible participants were adults (≥ 18 years) with a confirmed diagnosis of UC for at least 3 months. Patients must have completed at least 16 weeks of tofacitinib therapy for moderate to severe UC with complete clinical data or had discontinued treatment within the 16‐week induction period due to lack of response, adverse events, or other reasons. Available data up to 52 weeks after commencement of tofacitinib were collected. Patients with indeterminate colitis, acute severe ulcerative colitis (ASUC), Crohn's disease, prior colectomy including pouchitis, pregnancy, breastfeeding, recent venous thromboembolism (VTE) or solid organ malignancy within 5 years were excluded. Ethics approval for this research project has been granted for all study sites by Mater Misericordiae Ltd. Human Research Ethics Committee (HREC/MML/76990).

### 2.2 Outcomes and Definitions

The primary outcome for this study was clinical response to induction at week 8, with an extended induction response assessment at week 16 as per standard clinical practice. Clinical response was defined as a reduction in the partial Mayo score by at least 2 points from baseline or an overall partial Mayo score < 2 [11, 13]. Standard induction therapy was defined as 8 weeks of tofacitinib 10 mg twice daily, followed by a maintenance dose of 5 mg twice daily. Extended induction was defined as 16 weeks of tofacitinib 10 mg twice daily, followed by a maintenance dose of 5 mg or 10 mg twice daily. Patients who required reinduction with tofacitinib 10 mg twice daily following the initial induction period were included in the analysis and classified as treatment failures. Patients with treatment discontinuation for any reason, at any time during induction or subsequent maintenance therapy were included in the analysis and classified as treatment failures. Patients with moderate to severe UC (partial Mayo score ≥ 5) at baseline were included in this clinical response analysis.

The secondary endpoints at all timepoints include:

- clinical remission (defined as a partial or overall Mayo score ≤ 2 with no individual sub score greater than 1)
- resolution of rectal bleeding (Mayo rectal bleeding score = 0)
- endoscopic remission (defined as a Mayo endoscopic subscore ≤ 1 for those who underwent endoscopy within the follow up period)
- requirement for extended induction (16 weeks of tofacitinib twice daily)
- corticosteroid free clinical remission (CFCR, defined as clinical remission achieved without corticosteroid for at least 2 weeks prior to assessment)
- biochemical remission (defined as normalization of C‐reactive protein [CRP] and fecal calprotectin [FCP] level [≤ 5 mg/L and < 150 μg/g, respectively] in patients with baseline levels above these criteria)
- Rate of Colectomy during the study period

Safety outcomes included reported adverse events (AE) and serious adverse events (SAEs, defined as AE leading to drug discontinuation or hospitalization) at week 8, 16, and 52. Adverse events of special interest included serious and opportunistic infections; incidence and extent of herpes zoster (one or multiple dermatomes, ocular, disseminated disease), major adverse cardiovascular events (MACE), pulmonary embolism (PE), venous thromboembolism (VTE), non‐melanomatous skin cancer (NMSC), and malignancy.

### 2.3 Demographic and Clinical Data

Demographic and clinical data were collected by retrospective chart review. The following demographic data were collected: age at induction, sex, smoking history, age at diagnosis, disease duration, extent of disease, prior endoscopic severity, surgical history, presence of extra‐intestinal manifestations, prior and current medications including prior ADT exposure (defined as loss of response as per treating clinician with biologic or small molecule therapies). All prior IBD medications were documented, with current usage defined as any use within 8 weeks prior to tofacitinib induction. Concurrent medications, including corticosteroids and their cumulative doses, were documented throughout the follow‐up period.

Clinical, biochemical, and endoscopic data were collected at set time points: baseline (prior to commencement of tofacitinib), week 8 (+/− 14 days), week 16 (+/− 14 days) and week 52 (+/− 28 days) post commencement of tofacitinib therapy. All endoscopic assessments throughout the 52‐week follow‐up were collected, including both Mayo endoscopic subscore and Ulcerative Colitis Endoscopic Index of Severity (UCEIS) scores.

### 2.4 Statistical Analysis

Descriptive statistics were utilized for analysis of the primary and secondary outcomes. Data were taken as is, and no method of imputation was applied e.g., for missing data. Chi‐squared tests were used for subgroup comparison of categorical dichotomous outcomes of interest stratified by the need for extended induction. Baseline characteristics were expressed using descriptive statistics and Mann–Whitney *U* tests for non‐parametric continuous characteristics, expressed as median with interquartile ranges (IQRs). Note that while *p*‐values are reported for the sake of completeness, they should be considered descriptive statistics; no hypotheses are being tested. Analyses were conducted using “Statistical Package for the Social Sciences” (SPSS) version 29.

## 3 Results

A total of 124 patients met inclusion criteria and were included in the analysis. The clinical and demographic characteristics of the cohort are summarized in Table 1. The median age was 41 years (IQR 29–49), 41% were female, with a median disease duration of 9 years (IQR 5–14), with 6% actively smoking. Disease extent included isolated proctitis (E1) in 10% (*n* = 12), left‐sided colitis (E2) in 43% (*n* = 53), and extensive colitis (E3) in 47% (*n* = 59). The median baseline partial Mayo score was 5 (IQR 3–6).

**TABLE 1:** Baseline patient demographics.

Demographics, *n* (%)	Overall cohort (*n* = 124)	Standard induction (*n* = 50)	Extended induction (*n* = 74)	*p*
Female	51 (41)	25 (50)	26 (36)	0.07
Median age at time of induction, years (IQR)	41 (25–53)	46 (27–57)	35 (23–50)	0.09
Disease duration, years (IQR)	9 (3–11)	10 (3–12)	9 (4–11)	0.25
Montreal classification—disease extent				0.89
E1—proctitis	12 (10)	4 (8)	8 (11)	
E2—left‐sided UC	53 (43)	21 (43)	32 (43)	
E3—extensive UC	59 (47)	24 (49)	35 (47)	
Montreal classification—endoscopic disease severity				0.04
S1—mild	25 (20)	13 (27)	12 (18)	
S2—moderate	50 (40)	16 (32)	33 (45)	
S3—severe	49 (40)	21 (42)	29 (39)	
Current smoker	8 (6)	5 (10)	3 (4)	0.08
Number of prior advanced drug therapies				0.34
0	10 (8)	2 (4)	8 (10)	
1	44 (36)	18 (36)	26 (35)	
2	57 (46)	25 (50)	32 (42)	
≥ 3	13 (10)	6 (11)	7 (10)	
Prior advanced drug therapy				
Anti‐TNF	104 (84)	44 (89)	60 (80)	0.14
Anti‐integrin	76 (61)	32 (65)	44 (59)	0.42
Il‐12/23	22 (17)	9 (18)	13 (17)	0.85
Baseline concomitant medications				
Oral corticosteroid	35 (28)	14 (28)	21 (28)	0.92
Oral 5‐ASA	79 (63)	33 (67)	46 (62)	0.61
Median baseline CRP (IQR)	7 (2–17)	6 (2–15)	7 (2–14)	0.93
Median week 8 CRP (IQR)	4 (1–8)	4 (1–7)	5 (2–8)	0.90
Median baseline FCP (IQR)	645 (175–1219)	651 (145–1142)	632 (193–1371)	0.87
Median week 8 FCP (IQR)	171 (17–495)	196 (36–532)	160 (12–449)	0.71
Median baseline mayo endoscopic subscore (IQR)	2 (2–3)	2 (2–3)	2 (2–3)	0.95

Tofacitinib was used as a first‐line advanced drug therapy in 8% (*n* = 10) of patients, leaving 92% (*n* = 112) of patients exposed to at least 1 prior ADT. Of these, 36% (*n* = 44) had prior exposure to one ADT, 46% (*n* = 57) to two ADTs, and 10% (*n* = 13) to three or more ADTs. Drug specific ADT exposure included anti‐TNF therapy in 84% (*n* = 104), vedolizumab in 61% (*n* = 76), and ustekinumab in 18% (*n* = 22).

During induction, tofacitinib was given concomitantly with oral corticosteroids in 28% of patients, with oral aminosalicylates in 63%, and 21% received both corticosteroids and aminosalicylates. All patients received tofacitinib induction therapy of 10 mg twice daily for a minimum of 8 weeks. Following this initial induction period, 60% (74 of 124) continued with extended induction of 10 mg twice daily for an additional 8 weeks at the discretion of the treating clinician, while the remaining patients reduced to 5 mg twice daily (summarized in Table 1). While most patients transitioned to a maintenance dose of 5 mg twice daily for the remainder of the study, 5 patients who continued an escalated dose of 10 mg twice daily were excluded from the analysis.

### 3.1 Overall Clinical Effectiveness

**FIGURE 1:**
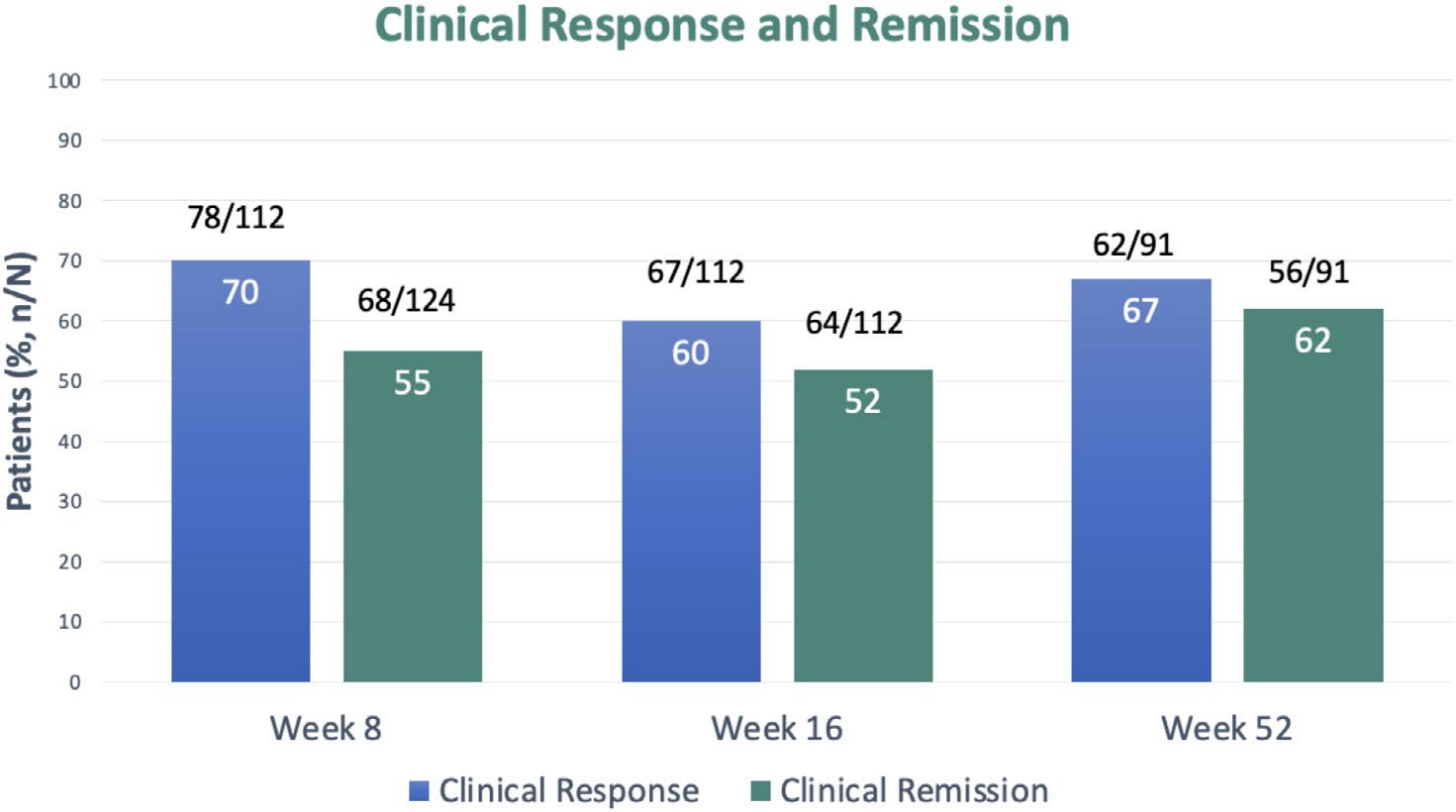
Overall clinical response and remission at week 8, 16 and 52.

Complete clinical data at both weeks 8 and 16 were available for all 124 patients, with 112 meeting criteria for moderate to severe disease at baseline and included in the clinical response analysis. The 12 excluded patients had mild to moderate disease but switched to tofacitinib therapy due to prior medication intolerance or patient and physician preference. The primary endpoint of clinical response was achieved by 70% (78 of 112) of patients at week 8, and 60% (67 of 112) at week 16 following tofacitinib initiation in patients with moderate to severe colitis activity at baseline (Figure 1). Clinical remission was observed in 55% (68 of 124) of patients at week 8 and 52% (64 of 124) at week 16.

At week 52, clinical data were available for 91 patients (73% of the cohort), with the remaining patients either lost to follow‐up (8%) or yet to complete 1 year of follow‐up (19%) at time of data collection. The 21 patients who discontinued tofacitinib during follow‐up as treatment failures were included using non‐response imputation. Clinical response rate was 67% (62 of 91) and remission rate was 62% (56 of 91) (Table 2).

**TABLE 2:** Primary and secondary outcomes.

Overall primary and secondary outcomes, *n*/*N* (%)	Baseline	Week 8	Week 16	Week 52
Clinical response	—	78/112 (70)	67/112 (60)	62/91 (68)
Clinical remission	12/124 (9)	68/124 (55)	64/124 (52)	56/91 (62)
Corticosteroid‐free clinical remission	—	16/35 (46)	14/35 (40)	13/20 (65)
Resolution of rectal bleeding	—	46/91 (50)	37/91 (41)	29/62 (47)
Resolution of stool frequency	—	22/109 (21)	28/109 (26)	19/72 (26)
Biochemical remission	41/117 (33)	44/76 (58)	46/76 (61)	28/54 (52)
Endoscopic healing	—	19/35 (54)	37/59 (63)	36/76 (47)
Colectomy rate	—	1/124 (1)	2/124 (2)	2/91 (3)

In the subset of 91 patients presenting with rectal bleeding at baseline, 50% (46 of 91) achieved complete resolution by week 8, 41% (37 of 91) by week 16, and 75% (47 of 63) by week 52. Of the 109 patients with increased stool frequency at baseline, 21% (23 of 109) had complete resolution by week 8, 26% (28 of 109) by week 16, and 59% (37 of 63) by week 52.

There were 35 patients receiving concomitant corticosteroid during induction. Among these, CFCR was achieved in 46% (16 of 35) at week 8 and 40% (14 of 35) patients at week 16. By week 52, no patients remained on corticosteroid, with an improvement in CFCR to 65% (13 of 20).

Twelve patients with ulcerative proctitis were treated with tofacitinib, with 83% (10 of 12) achieving clinical response by week 8 and 92% (11 of 12) by week 16. Clinical remission rates were also high in this cohort at 58% at week 8 (7 of 12) and 83% (10 of 12) by week 16. Clinical response (73%) and remission rates (73%) remained high throughout 52 weeks of follow‐up.

### 3.2 Standard Versus Extended Induction

Among the 50 patients who received standard induction, 70% (35 of 50) achieved clinical response and 58% (29 of 50) achieved clinical remission at week 8. By week 16 (after 8 weeks of dose reduction to 5 mg twice daily), only 46% (23 of 50) met criteria for clinical response and 44% (22 of 50) for clinical remission. By week 52, data were available for 35 of those 50 patients; 54% (19 of 35) met criteria for clinical response and 53% (18 of 35) for clinical remission.

Among the 74 patients who received extended induction, 70% (52 of 74) achieved clinical response and 53% (39 of 74) achieved clinical remission at week 8. By week 16, 69% (51 of 74) met criteria for clinical response and 55% (41 of 74) for clinical remission. By week 52, data were available for 55 of those 74 patients; 75% (41 of 55) met criteria for clinical response and 67% (37 of 55) for clinical remission.

When considering only those patients meeting criteria for clinical response at week 8 (70%) of both cohorts, the standard induction cohort saw a 40% (14 of 35) loss of clinical response by week 16, with 46% (16 of 35) of these meeting criteria for clinical remission. In comparison, the extended induction cohort saw only 6% (3 of 52) loss of clinical response in the same timeframe, with 73% (38 of 52) of these meeting criteria for clinical remission. In patients with clinical response at week 8, extended induction with 16 weeks of tofacitinib 10 mg BD resulted in significantly improved maintenance of clinical response compared with standard induction (94% vs. 60%, *p* ≤ 0.01) and higher rates of clinical remission (73% vs. 46%, *p* ≤ 0.01).

**FIGURE 2:**
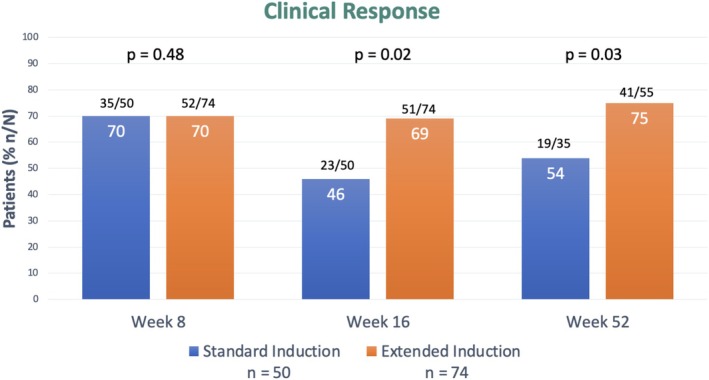
Clinical response at week 8, 16 and 52 stratified by duration of induction.

These two cohorts were well matched in general, but the extended induction cohort had a significantly higher endoscopic disease severity at baseline (*p* = 0.04) (Table 1). While there was no difference in the response rates between cohorts at week 8 (70% vs. 70%, *p* = 0.48), a significant increase in the rate of clinical response for extended induction was seen at both week 16 (69% vs. 46%, *p* = 0.02) and 52 (75% vs. 54%, *p* = 0.03) compared to standard induction (Figure 2). Although clinical remission rates were also higher with extended induction, this difference did not reach statistical significance at week 16 (55% vs. 44%, *p* = 0.25) or 52 (67% vs. 53%, *p* = 0.22) (Figure 2). There were no significant predictors of response apart from duration of induction therapy when considering baseline clinical, biochemical and demographic status in either cohort.

### 3.3 Biochemical Outcomes

Complete biochemical data at baseline and within the first 16 weeks of follow up were available for 117 patients (Table 2). The median CRP level decreased from 7 mg/L (IQR 2–17) at baseline to 4 mg/L (IQR 1–8) at week 8, and further to 2 mg/L (IQR 1–7) at week 16. The median FCP level also decreased from 645 μg/g (IQR 175–1440) at baseline to 171 μg/g (IQR 17–495) at week 8, and further to 143 μg/g (IQR 24–346) at week 16. At baseline, 35% (41 of 117) met criteria for biochemical remission. Of those with significantly elevated FCP or CRP at baseline, 58% (44 of 76) achieved biochemical remission at week 8, 61% (46 of 76) by week 16 and 54% (26 of 48) by week 52.

In the standard induction cohort, 63% (20 of 32) achieved biochemical remission at week 8, 56% (18 of 32) at week 16, and 55% (12 of 22) at week 52. In the extended induction cohort, 56% (25 of 44) achieved biochemical remission at week 8, 68% (30 of 44) at week 16, and 54% (14 of 26) at week 52. There was no significant difference in the rate of biochemical remission at any timepoint between the cohorts (week 8 *p* = 0.34, week 16 *p* = 0.15, week 52 *p* = 0.43).

### 3.4 Endoscopic Outcomes

Among the 93 patients who underwent baseline endoscopy within 1 month of tofacitinib induction, 59 had a repeat endoscopy at week 16 and 76 at week 52. The baseline median Mayo endoscopic subscore was 2 (IQR 2–3), which reduced to 1 (IQR 0–1) at week 16 and 1 (IQR 0–2) at week 52. Endoscopic remission (MES of 0–1) was observed in 63% (37 of 59) of patients at week 16 and 47% (36 of 76) at week 52.

In the standard induction cohort, 54% (15 of 28) achieved endoscopic remission at week 16, and 41% (14 of 34) at week 52. In the extended induction cohort, 68% (21 of 31) achieved endoscopic remission at week 16, and 50% (21 of 42) at week 52. There was no significant difference in the rate of biochemical remission at any timepoint between the cohorts (week 16 *p* = 0.09, week 52 *p* = 0.21).

### 3.5 Safety and Adverse Events

Over the 52 week follow up period, AEs were reported in 17% (21 of 124) of patients, collated in Table 3. The most common AEs were acne (5%), herpes zoster (5%) and headache (4%). No significant difference was seen in the rate of AEs in those receiving standard vs. extended induction (14% vs. 18%, *p* = 0.32), although over half (63%) of patients who experienced AEs occurred within the first 8 or 16 weeks while on the induction dose of 10 mg BD and resolved after dose reduction. There were no reports of drug interruption or discontinuation due to AEs within the initial 8 or 16 week induction period, although 6 patients ultimately discontinued tofacitinib due to AEs and were counted as SAEs.

**TABLE 3:** Adverse events (AEs), serious adverse events (SAEs) and adverse events of special interest (AESIs).

Adverse event, *n* patients (%)	Overall cohort (*n* = 124)	Standard induction (*n* = 50)	Extended induction (*n* = 74)
Total adverse events	21 (17)	8 (16)	13 (18)
Acne	6 (5)	2 (4)	4 (5)
Herpes zoster infection	6 (5)	2 (4)	4 (5)
Headache	5 (4)	2 (4)	3 (4)
Nasopharyngitis	4 (3)	2 (4)	2 (3)
Patients with Multiple adverse events	5 (4)	2 (4)	3 (4)
Serious adverse events	9 (7)	4 (8)	5 (7)
Severe infection	1 (1)	0 (0)	1 (2)
Colectomy (after ASUC)	2 (2)	1 (2)	1 (2)
Drug discontinuation due to AE	6 (5)	3 (6)	3 (4)
Adverse events of special interest	8 (6)	3 (6)	5 (7)
Serious infections	1 (1)	0 (0)	1 (2)
Opportunistic infections	0 (0)	0 (0)	0 (0)
Herpes zoster	6 (5)	2 (4)	4 (5)
MACE	0 (0)	0 (0)	0 (0)
NMSC	1 (1)	1 (2)	0 (0)
Malignancy	0 (0)	0 (0)	0 (0)
VTE	0 (0)	0 (0)	0 (0)

SAEs were reported in 7% (9/124) of patients, with 6 requiring discontinuation of drug and 3 requiring hospital admission. The most common reason for drug discontinuation in these patients was recurrent herpes zoster infection (50%, 3/6), where the patients had not received the shingles vaccine. Two required hospital admission for intravenous corticosteroid therapy due to ASUC, attributed to lack of efficacy with disease progression rather than a drug‐related side effect. The third admission was for the management of *Clostridium difficile* infection. During follow up, two patients underwent colectomy due to lack of response to tofacitinib.

AESI were reported in 6% (8/124) of patients, collated in Table 3. Six patients suffering herpes zoster infections, 1 serious infection with *Clostridium difficile* and 1 case of non‐melanomatous skin cancer (NMSC) were reported, with no reported episodes of MACE, VTE, malignancy or death.

## 4 Discussion and Conclusions

This study presents the largest Australian cohort to date evaluating the real‐world outcomes of tofacitinib in ulcerative colitis. Our findings confirm that tofacitinib is effective for induction of clinical response, clinical remission and CFCR in a real‐world setting, with response rates higher than those reported in clinical trials. Clinical response rates of 70% by week 8, 60% by week 16, and 62% by week 52 in our cohort may reflect differences in the definition of clinical remission, as we used the partial Mayo score (a clinical score incorporating the subjective physician global assessment) rather than the total Mayo score including endoscopic subscore, which was used in the phase 3 OCTAVE trials. Other potential factors may relate to the uncontrolled nature of real‐world practices compared to the strict inclusion and exclusion criteria used in the registration clinical trials.

When comparing these results with published real world data from a similarly sized cohort in the United Kingdom, we confirm similarly high rates of clinical response (using the same partial mayo score criteria for response) at week 8 (74% vs. 70%) and 16 (66% vs. 60%), although in the study by Honap et al. the response rates were not assessed by duration of induction therapy [11]. Ma et al. reported significantly lower rates of clinical remission post‐induction (35% vs. 52%) and by week 52 (35% vs. 62%), along with lower rates of endoscopic remission in a large Canadian cohort treated with tofacitinib [10]. That cohort had a higher rate of corticosteroid use during induction therapy, higher initial CRP levels, higher proportion of patients with ≥ 2 ADT failures with over 10% of patients being hospitalized during or immediately after induction, suggesting more severe disease at baseline than our cohort.

We have shown that patients undergoing extended induction with 16 weeks of tofacitinib 10 mg twice daily have significantly higher rates of clinical response at week 16 compared to those with a standard 8 week induction regimen (69% vs. 45%, *p* = 0.02), with sustained response through the first year of therapy (75% vs. 54%, *p* = 0.03). This occurred despite the cohort receiving extended induction having more severe disease endoscopically at baseline (*p* = 0.04), and all patients continuing tofacitinib 5 mg twice daily post induction, differing from the study by Tzouvala et al. where one cohort continued 10 mg twice daily dosing for a year of follow‐up [12]. When comparing these cohorts at week 8, there was no significant difference in clinical response or remission (*p* = 0.51), which is unsurprising given both had received identical therapy with induction of 8 weeks of tofacitinib 10 mg twice daily at that point. In the Octave sustain trial, patients with clinical response at week 8 of induction were randomized to either standard or extended induction for a further 52 weeks. Both standard and extended induction regimes were significantly superior to placebo in terms of clinical remission at week 52, but no significant difference was seen when comparing with each other (34.3% vs. 40.6%). In this study, focusing only on the patients with clinical response at week 8 in a similar fashion to Octave sustain, we saw extended induction was associated with significantly higher rates of maintenance of clinical response (94% vs. 60%, *p* ≤ 0.01) and clinical remission (73% vs. 46%, *p* ≤ 0.01) at week 16 compared to standard induction. This differs from the results seen in Octave sustain, suggesting even patients with clinical response at week 8 may benefit from an extended induction with 16 weeks of tofacitinib 10 mg BD. While interesting, there are a number of potential explanations for this difference between studies, the most likely being the difference in timing of outcome (16 vs. 52 weeks) and differing definitions of both clinical response and remission.

While rates of overall clinical remission, biochemical remission and CFCR were all numerically higher in the extended induction cohort at weeks 16 and 52, these did not meet criteria for statistical significance. The decision to pursue standard or extended induction was made entirely at the discretion of the treating physician, without any standardized criteria for deciding duration of induction considering baseline criteria or degree of response by week 8. As a result, we cannot deduce or comment if one cohort of patients may benefit most from an extended induction regime, or if this benefit extends to the entire cohort.

A notable finding in this study was the overall reduction in clinical response (70%–60%) and remission rates (55%–52%) between week 8 and 16. It appears that the standard induction cohort accounts for the majority of the observed reduction in clinical effectiveness. The rate of clinical response in the extended induction cohort was similar at 70% at week 8 and 69% at week 16. However, clinical response in the standard induction cohort dropped from 71% at week 8 to 45% at week 16.

These data are consistent with the established safety profile with tofacitinib reported in clinical trials and real world data. The most common AEs were mild and self‐limiting, including acne, herpes zoster, and nasopharyngitis, primarily observed in patients while undergoing induction therapy with tofacitinib 10 mg twice daily. Importantly, no cases of VTE, MACE, or malignancies (excluding NMSC) were observed, although the relatively short follow‐up period of 1 year limits the detection of rarer, long‐latency events.

This study has limitations inherent to its retrospective design, including the potential for recall bias which, although mitigated by the inclusion of all consecutively treated patients, is an unavoidable risk. The small cohort size and post hoc nature of analysis may add further bias. Additionally, the limited availability of follow up endoscopic and biochemical data limited our ability to fully evaluate response durability. Potentially the most significant limitation relates to the decision regarding standard or extended induction dosing being at the discretion of the treating physician, which may introduce further selection bias and limit causal interpretation. Variability in treatment practices across centers and clinician discretion in dosing and monitoring may also influence outcomes, making it challenging to directly generalize results across different healthcare settings.

In conclusion, tofacitinib is an effective and safe therapy in a real‐world Australian population with moderate to severe ulcerative colitis, demonstrating high rates of clinical and corticosteroid free remission. Extended induction therapy with 10 mg twice daily for 16 weeks resulted in higher clinical response rates observed at week 16, maintained through week 52.

## Funding

This study was sponsored by Pfizer.

## Conflicts of Interest

Y.A. reports grants from Janssen, during the conduct of the study; has received speaking and consulting fees from Abbvie, Bristol Myers Squibb, Celltrion, Chiesi, Dr. Falk, Ferring, Janssen, Pfizer, Sandoz, Shire and Takeda; served as an advisory board member for Abbvie, Bristol Myers Squibb, Chiesi, Janssen, NPS Medicine wise, Microba; received research and educational funding from Abbvie, Celltrion, Dr. Falk, Janssen, Pfizer, Sandoz and Takeda.

J.B. has received speaking and consulting fees from Abbvie, Bristol Myers Squibb, Celltrion, Chiesi, Dr. Falk, Ferring, Janssen, Pfizer, Sandoz, Shire, and Takeda; served on advisory boards member for Abbvie, Bristol Myers Squibb, Chiesi, Janssen, NPS Medicine wise, Anatara, Microba; received research and educational funding from Abbvie, Janssen, Pfizer, and Takeda.

L.T. has received funding from Pfizer, Takeda, Celltrion, advisory board fees from Abbvie, Janssen, Pfizer, Celltrion, BMS, Sandoz, speaker fees from Abbvie, Janssen, Pfizer, Celltrion, Takeda, BMS, Dr. FALK.

M.G. has served on the advisory board of Abbvie, Pfizer and Ferring, and has received speaker fees, research or travel grants from Abbvie, Celltrion, Dr. Falk, Ferring, Fresenius Kabi, Janssen, Pfizer, Pharmacosmos, Takeda.

M.P.S. has received educational grants or research support from Gilead and Celltrion, speakers fees from Janssen, Abbvie, Ferring, Takeda, Pfizer, Shire, Celltrion, Eli‐Lilly, and Dr. Falk Pharma, and has served on advisory boards or received consultancy fees from Janssen, Takeda, Pfizer, Celgene, Abbvie, MSD, Emerge Health, Gilead, BMS, Celltrion, Eli‐Lilly, and Alimentiv.

E.K.W. has received educational grants or research support from AbbVie and Ferring and speaker fees/consulting from Janssen, Abbvie, BMS, Ferring, Takeda, Pfizer, Celltrion, and Dr. Falk Pharma, and has served on advisory boards or received consultancy fees from Janssen, Abbvie, and Ferring.

S.G. has received educational or research support from Ferring and speaker fees/consulting fees from Janssen, Takeda, Abbvie, BMS, Ferring, Pfizer, and Dr. Falk pharma.

G.T.M. has received educational or research support from AbbVie, Janssen, Pfizer, Shire, and Takeda, speaker fees from AbbVie, Falk, Ferring, Janssen, MSD, Orphan, Pfizer, Roche, Sandoz, Shire, and Takeda, and has served on advisory boards for AbbVie, Celltrion, Emerge, Eli Lilly, Gilead, Hospira, Janssen, MSD, Orphan, Pfizer, Shire, and Takeda.

M.G.W. has received educational grants and speakers fees from AbbVie, Takeda, Janssen and Ferring; travel grants from Pfizer and Dr. Falk; and has served on advisory boards for AbbVie and Janssen.

R.G.F. has received grant support from the Gastroenterology Network of Intestinal Ultrasound (GENIUS) and Crohn's and Colitis Australia (CCA), and speaker, advisory board or consultation honoraria from Janssen, AbbVie and Ferring, and education or conference support from Ferring, Pfizer, AbbVie, Falk, Janssen, Celltrion, BMS.

P.D.C. has served as a consultant, an advisory board member, or a speaker for AbbVie, Baxter, Ferring, Janssen, Celltrion, Pfizer, Emerge Health, Shire, and Takeda and has received research support from AbbVie, Ferring, Shire, Janssen, Pfizer, and Takeda.

A.T. and C.d.F.P. are employees and stockholders of Pfizer. The other authors declare no conflicts of interest.

## Data Availability

The data that support the findings of this study are available on request from the corresponding author. The data are not publicly available due to privacy or ethical restrictions.

## References

[1] C. Lin , F. Cooles , and J. Isaacs , “Basic Mechanisms of Jak Inhibition,” Mediterranean Journal of Rheumatology 31 (2020): 100. 32676567 10.31138/mjr.31.1.100 PMC7361186

[2] W. Sandborn , C. Su , B. Sands , et al., “Tofacitinib as Induction and Maintenance Therapy for Ulcerative Colitis,” New England Journal of Medicine 376 (2017): 1723–1736. 28467869 10.1056/NEJMoa1606910

[3] W. Sandborn , L. Peyrin‐Biroulet , D. Quirk , et al., “Efficacy and Safety of Extended Induction With Tofacitinib for the Treatment of Ulcerative Colitis,” Clinical Gastroenterology and Hepatology: The Official Clinical Practice Journal of the American Gastroenterological Association 20 (2022): 1821–1830.e3. 33127596 10.1016/j.cgh.2020.10.038

[4] W. Sandborn , N. Lawendy , S. Danese , et al., “Safety and Efficacy of Tofacitinib for Treatment of Ulcerative Colitis: Final Analysis of Octave Open, an Open‐Label, Long‐Term Extension Study With up to 7.0 Years of Treatment,” Alimentary Pharmacology & Therapeutics 55 (2022): 464–478. 34854095 10.1111/apt.16712 PMC9300081

[5] P. Mease , C. Charles‐Schoeman , S. Cohen , et al., “Incidence of Venous and Arterial Thromboembolic Events Reported in the Tofacitinib Rheumatoid Arthritis, Psoriasis and Psoriatic Arthritis Development Programmes and From Real‐World Data,” Annals of the Rheumatic Diseases 79 (2020): 1400–1413. 32759265 10.1136/annrheumdis-2019-216761 PMC7569391

[6] S. Ytterberg , D. Bhatt , T. Mikuls , et al., “Cardiovascular and Cancer Risk With Tofacitinib in Rheumatoid Arthritis,” New England Journal of Medicine 386 (2022): 316–326. 35081280 10.1056/NEJMoa2109927

[7] R. Gilmore , P. Hilley , A. Srinivasan , M. Choy , and P. De Cruz , “Sequential Use of High‐Dose Tofacitinib After Infliximab Salvage Therapy in Acute Severe Ulcerative Colitis,” Journal of Crohn's & Colitis 16 (2022): 166–168. 10.1093/ecco-jcc/jjab109 34159363

[8] J. Berinstein , J. Sheehan , M. Dias , et al., “Tofacitinib for Biologic‐Experienced Hospitalized Patients With Acute Severe Ulcerative Colitis: A Retrospective Case‐Control Study,” Clinical Gastroenterology and Hepatology: The Official Clinical Practice Journal of the American Gastroenterological Association 19 (2021): 2112–2120. 34048936 10.1016/j.cgh.2021.05.038 PMC8760630

[9] A. Singh , M. Goyal , V. Midha , et al., “Tofacitinib in Acute Severe Ulcerative Colitis (Tacos): A Randomized Controlled Trial,” American Journal of Gastroenterology 119 (2024): 1365–1372. 38131615 10.14309/ajg.0000000000002635

[10] C. Ma , R. Panaccione , Y. Xiao , et al., “Remit‐Uc: Real‐World Effectiveness and Safety of Tofacitinib for Moderate‐To‐Severely Active Ulcerative Colitis: A Canadian Ibd Research Consortium Multicenter National Cohort Study,” American Journal of Gastroenterology 118 (2023): 861–871. 36580497 10.14309/ajg.0000000000002129 PMC10144270

[11] S. Honap , D. Chee , T. Chapman , et al., “Real‐World Effectiveness of Tofacitinib for Moderate to Severe Ulcerative Colitis: A Multicentre UK Experience,” Journal of Crohn's & Colitis 14 (2020): 1385–1393. 10.1093/ecco-jcc/jjaa075 32280965

[12] M. Tzouvala , E. Zacharopoulou , M. Kalafateli , et al., “Value of Tofacitinib Extended Induction Therapy in Patients With Moderate‐To‐Severe Ulcerative Colitis: A Real‐World 52‐Week Follow‐Up Study,” World Journal of Gastroenterology 31 (2025): 111282. 41278164 10.3748/wjg.v31.i42.111282 PMC12635784

[13] G. D'Haens , W. Sandborn , B. Feagan , et al., “A Review of Activity Indices and Efficacy End Points for Clinical Trials of Medical Therapy in Adults With Ulcerative Colitis,” Gastroenterology 132 (2007): 763–786. 17258735 10.1053/j.gastro.2006.12.038

